# *N*-{3-[Bis(2-hydroxy­ethyl)amino­meth­yl]-5-nitro­phen­yl}benzamide

**DOI:** 10.1107/S1600536808009410

**Published:** 2008-05-03

**Authors:** Anna Mai, Gul S. Khan, George R. Clark, David Barker

**Affiliations:** a Department of Chemistry, The University of Auckland, Private Bag 92019, Auckland, New Zealand

## Abstract

The title compound, C_18_H_21_N_3_O_5_, was prepared by the reaction of 3-benzamido-5-nitro­benzyl methane­sulfonate with diethano­lamine and is an inter­mediate in the synthesis of DNA minor-groove-binding polybenzamide agents capable of being conjugated to additional biologically active species. The asymmetric unit contains two independent mol­ecules, which differ only in the orientations of the hydroxy­ethyl groups. In the crystal structure, inter­molecular N—H⋯O and O—H⋯O hydrogen bonds link mol­ecules into one-dimensional chains.

## Related literature

For related literature on the biological activity of polybenzamide DNA binding agents, see: Storl *et al.* (1993 ▶). For related literature on natural and synthetic minor-groove binding agents, including agents containing conjugates, see: Arcamone *et al.* (1964 ▶); Atwell *et al.* (1995 ▶); Baraldi *et al.* (1999 ▶, 2004 ▶, 2007 ▶); Kumar *et al.* (2004 ▶); Sengupta *et al.* (1996 ▶); Stafford *et al.* (2007 ▶); Turner *et al.* (1999 ▶); Wemmer (2000 ▶); Yan *et al.* (1997 ▶). For related literature, see: Barker *et al.* (2008 ▶).
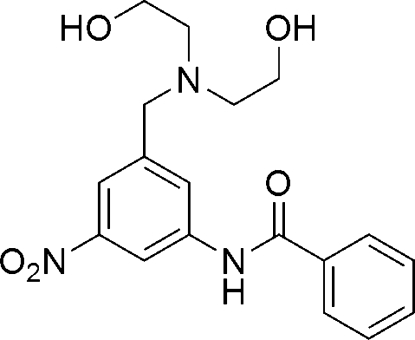


## Experimental

### 

#### Crystal data

C_18_H_21_N_3_O_5_
*M* *_r_* = 359.38
Monoclinic, 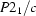

*a* = 22.7867 (3) Å
*b* = 11.0879 (1) Å
*c* = 13.5106 (1) Å
β = 90.114 (1)°
*V* = 3413.54 (6) Å^3^
*Z* = 8
Mo *K*α radiation
μ = 0.10 mm^−1^
*T* = 90 (2) K
0.34 × 0.22 × 0.20 mm

#### Data collection

Bruker SMART CCD diffractometer
Absorption correction: multi-scan (*SADABS*; Sheldrick, 1997 ▶) *T*_min_ = 0.858, *T*_max_ = 0.978
20279 measured reflections
6944 independent reflections
5142 reflections with *I* > 2σ(*I*)
*R*_int_ = 0.039

#### Refinement

*R*[*F* ^2^ > 2σ(*F* ^2^)] = 0.058
*wR*(*F* ^2^) = 0.134
*S* = 1.04
6944 reflections
485 parameters
H atoms treated by a mixture of independent and constrained refinement
Δρ_max_ = 0.25 e Å^−3^
Δρ_min_ = −0.29 e Å^−3^

### 

Data collection: *SMART* (Bruker, 1995 ▶); cell refinement: *SAINT* (Bruker, 1995 ▶); data reduction: *SAINT*; program(s) used to solve structure: *SHELXS97* (Sheldrick, 2008 ▶); program(s) used to refine structure: *SHELXL97* (Sheldrick, 2008 ▶); molecular graphics: *ORTEPIII* (Burnett & Johnson, 1996 ▶); software used to prepare material for publication: *SHELXTL* (Sheldrick, 2008 ▶).

## Supplementary Material

Crystal structure: contains datablocks I, global. DOI: 10.1107/S1600536808009410/lh2602sup1.cif

Structure factors: contains datablocks I. DOI: 10.1107/S1600536808009410/lh2602Isup2.hkl

Additional supplementary materials:  crystallographic information; 3D view; checkCIF report

## Figures and Tables

**Table 1:** Hydrogen-bond geometry (Å, °)

*D*—H⋯*A*	*D*—H	H⋯*A*	*D*⋯*A*	*D*—H⋯*A*
O4*A*—H*O*4*A*⋯O1*A*^i^	0.93 (4)	1.82 (4)	2.736 (2)	168 (3)
O5*A*—H*O*5*A*⋯O4*B*	0.89 (3)	1.85 (3)	2.738 (2)	178 (3)
N1*A*—H1*A*⋯O5*B*	0.86	2.27	3.089 (2)	159
O5*B*—H*O*5*B*⋯O4*B*	0.90 (4)	2.30 (4)	3.130 (3)	153 (3)
O4*B*—H*O*4*B*⋯O4*A*	1.03 (4)	1.75 (4)	2.762 (3)	169 (3)
N1*B*—H1*B*⋯O5*A*	0.86	2.49	3.332 (2)	167

Symmetry code: (i) 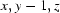.

**Fig. 1.**
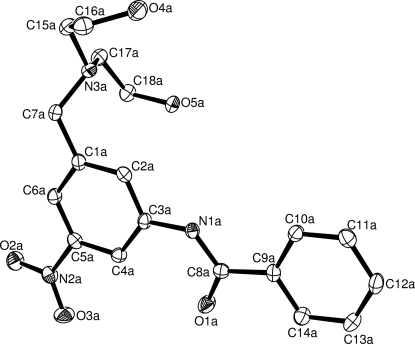
Molecular structure of one of the independent molecules showing 50% probability displacement ellipsoids for non-hydrogen atoms and hydrogen atoms as arbitary spheres (Burnett & Johnson, 1996).

## supplementary crystallographic information

### Comment

The naturally occurring antibiotic oligopeptides distamycin A, isolated from *Streptomyces Distallicus*, and netropsin, from *Streptomyces netropsis*, are powerful DNA minor groove-binding agents but their cytotoxity precludes their use as medicines (Arcamone *et al.*, 1964, Baraldi *et al.*, 2004, Wemmer *et al.*, 2000, Storl *et al.*, 1993). In order to increase the DNA binding affinity and sequence specificity along with minimizing the unwanted physiological activities associated with these natural DNA binders, many synthetic oligopeptides have been prepared (Baraldi *et al.*, 2007). In addition, numerous biologically active species have been conjugated to natural and synthetic DNA binding oligopeptides with the purpose of increasing the concentration of these species near DNA (Kumar *et al.*, 2004, Stafford *et al.*, 2007). The title compound is a key intermediate required in the synthesis of a novel polybenzamide DNA minor groove-binding agent.

### Experimental

*N*,*N*-Bis(2-hydroxyethyl)-3-benzamido-5-nitrobenzylamine was prepared using the method of Barker *et al.*(2008). To a solution of 3-benzamido-5-nitrobenzyl methanesulfonate (0.129 g, 0.368 mmol) in dry THF (1 ml) was added dropwise to a stirred suspension of diethanolamine (0.387 g, 3.68 mmol) in dry THF (2 ml) at 273 K. The mixture was then stirred under an atmosphere of nitrogen overnight before being concentrated *in vacuo* to give a crude residue. This residue was diluted with ethyl acetate (10 ml) and extracted with 2*M* HCl (2 *x* 10 ml). The combined acidic extracts were neutralized with 4*M* NaOH and then extracted with ethyl acetate (2 *x* 15 ml). The combined organic extracts were dried (MgSO_4_), filtered and the solvent removed *in vacuo*, to afford the title compound (0.128 g, 97%), as a yellow solid, which was recrystallized from ethyl acetate to give yellow crystals (m.p. 385–387 K) suitable for X-ray crystallography. Spectroscopic analysis: IR (ν_max_, thin film, cm^-1^) 2906, 1680, 1527, 1377. ^1^H NMR (300 MHz, CDCl_3_, δ, p.p.m.) 2.60 (4*H*, m, N(CH_2_CH_2_OH)_2_, 3.61 (2*H*, s, Ar—CH_2_N), 3.68 (4*H*, m, N(CH_2_CH_2_OH)_2_), 7.32 (3*H*, m, Ar—H), 7.46 (1*H*, m, Ar—H), 7.59 (1*H*, s, Ar—H), 7.67 (1*H*, m, Ar—H), 8.10 (1*H*, br s, Ar—H), 8.35 (1*H*, m, Ar—H) and 9.03 (NH). ^13^C NMR (75 MHz, CDCl_3_, δ, p.p.m.) 55.9 (CH_2_, N(*C*H_2_CH_2_OH)_2_), 58.4 (CH_2_, Ar—CH_2_N), 59.2 (CH_2_, N(CH_2_*C*H_2_OH)_2_), 114.5 (CH, Ar—C), 118.6 (CH, Ar—C), 125.9 (CH, Ar—C), 127.2 (CH, Ar—C), 128.4 (CH, Ar—C), 132.3 (CH, Ar—C), 133.2 (quat. Ar—C), 139.2 (quat. Ar—C), 142.3 (quat. Ar—C), 148.1 (quat. Ar—C) and 166.4 (C=O) MS m/*z* (FAB) 360 (*M*^+^, 9%), 219 (4), 154 (100), and 120 (NHCOC_6_H_5_, 8). HRMS (FAB), found: MH^+^ 360.15572. C_18_H_22_N_3_O_5_ requires: 360.15595.

### Refinement

Most hydrogen atoms were placed in calculated positions and refined using the riding model with C—H 0.93–0.97 Å and N—H = 0.86 Å, with *U*_iso_(H) = 1.2 or 1.5 times *U*_eq_(C). H atoms bonded to O atoms were located in a difference map and refined independently with isotropic displacement parameters.

### Crystal data

**Table:**

C_18_H_21_N_3_O_5_	*F*_000_ = 1520
*M**_r_* = 359.38	*D*_x_ = 1.399 Mg m^−^^3^
Monoclinic, *P*2_1_/*c*	Mo *K*α radiation λ = 0.71073 Å
Hall symbol: -P 2ybc	Cell parameters from 8192 reflections
*a* = 22.7867 (3) Å	θ = 0.9–26.4º
*b* = 11.0879 (1) Å	µ = 0.10 mm^−^^1^
*c* = 13.5106 (1) Å	*T* = 90 (2) K
β = 90.114 (1)º	Needle, yellow
*V* = 3413.54 (6) Å^3^	0.34 × 0.22 × 0.20 mm
*Z* = 8	

### Data collection

**Table:**

Bruker SMART CCD diffractometer	6944 independent reflections
Radiation source: fine-focus sealed tube	5142 reflections with *I* > 2σ(*I*)
Monochromator: graphite	*R*_int_ = 0.039
*T* = 90(2) K	θ_max_ = 26.4º
area–detector ω scans	θ_min_ = 0.9º
Absorption correction: multi-scan(SADABS; Sheldrick, 1997)	*h* = −28→25
*T*_min_ = 0.858, *T*_max_ = 0.978	*k* = −12→13
20279 measured reflections	*l* = −16→16

### Refinement

**Table:**

Refinement on *F*^2^	Secondary atom site location: difference Fourier map
Least-squares matrix: full	Hydrogen site location: inferred from neighbouring sites
*R*[*F*^2^ > 2σ(*F*^2^)] = 0.058	H atoms treated by a mixture of independent and constrained refinement
*wR*(*F*^2^) = 0.134	*w* = 1/[σ^2^(*F*_o_^2^) + (0.0471*P*)^2^ + 3.1255*P*] where *P* = (*F*_o_^2^ + 2*F*_c_^2^)/3
*S* = 1.04	(Δ/σ)_max_ < 0.001
6944 reflections	Δρ_max_ = 0.25 e Å^−^^3^
485 parameters	Δρ_min_ = −0.29 e Å^−^^3^
Primary atom site location: structure-invariant direct methods	Extinction correction: none

### Special details

**Table:**

Geometry. All e.s.d.'s (except the e.s.d. in the dihedral angle between two l.s. planes) are estimated using the full covariance matrix. The cell e.s.d.'s are taken into account individually in the estimation of e.s.d.'s in distances, angles and torsion angles; correlations between e.s.d.'s in cell parameters are only used when they are defined by crystal symmetry. An approximate (isotropic) treatment of cell e.s.d.'s is used for estimating e.s.d.'s involving l.s. planes.
Refinement. Refinement of *F*^2^ against ALL reflections. The weighted *R*-factor *wR* and goodness of fit *S* are based on *F*^2^, conventional *R*-factors *R* are based on *F*, with *F* set to zero for negative *F*^2^. The threshold expression of *F*^2^ > σ(*F*^2^) is used only for calculating *R*-factors(gt) *etc*. and is not relevant to the choice of reflections for refinement. *R*-factors based on *F*^2^ are statistically about twice as large as those based on *F*, and *R*- factors based on ALL data will be even larger.

### Fractional atomic coordinates and isotropic or equivalent isotropic displacement parameters (Å^2^)

**Table:**

	*x*	*y*	*z*	*U*_iso_*/*U*_eq_	
O1A	0.75115 (7)	0.40282 (14)	0.38171 (13)	0.0273 (4)	
O2A	0.49791 (7)	0.27802 (15)	0.31865 (13)	0.0281 (4)	
O3A	0.56436 (7)	0.41672 (15)	0.30441 (13)	0.0313 (4)	
O4A	0.74524 (7)	−0.35100 (16)	0.37188 (13)	0.0298 (4)	
HO4A	0.7493 (15)	−0.433 (3)	0.384 (2)	0.066 (11)*	
O5A	0.67682 (7)	−0.03575 (15)	0.58394 (12)	0.0220 (4)	
HO5A	0.7008 (14)	−0.098 (3)	0.574 (2)	0.051 (9)*	
N1A	0.75628 (7)	0.19893 (16)	0.37317 (13)	0.0169 (4)	
H1A	0.7800	0.1387	0.3734	0.020*	
N2A	0.54971 (8)	0.31179 (18)	0.31848 (14)	0.0218 (4)	
N3A	0.63691 (8)	−0.19644 (16)	0.41918 (13)	0.0186 (4)	
C1A	0.62210 (9)	0.0133 (2)	0.35151 (15)	0.0185 (5)	
C2A	0.68029 (9)	0.0499 (2)	0.36241 (15)	0.0176 (5)	
H2A	0.7094	−0.0081	0.3708	0.021*	
C3A	0.69585 (9)	0.1717 (2)	0.36096 (15)	0.0167 (5)	
C4A	0.65270 (9)	0.2599 (2)	0.34618 (15)	0.0179 (5)	
H4A	0.6621	0.3415	0.3440	0.021*	
C5A	0.59536 (9)	0.2201 (2)	0.33495 (15)	0.0186 (5)	
C6A	0.57860 (9)	0.1005 (2)	0.33811 (15)	0.0179 (5)	
H6A	0.5394	0.0786	0.3315	0.022*	
C7A	0.60623 (9)	−0.1185 (2)	0.34772 (16)	0.0199 (5)	
H7A1	0.5643	−0.1261	0.3587	0.024*	
H7A2	0.6143	−0.1482	0.2816	0.024*	
C8A	0.78069 (9)	0.3098 (2)	0.38447 (15)	0.0178 (5)	
C9A	0.84602 (9)	0.3155 (2)	0.39933 (14)	0.0170 (5)	
C10A	0.88212 (9)	0.2143 (2)	0.40848 (15)	0.0188 (5)	
H10A	0.8659	0.1374	0.4061	0.023*	
C11A	0.94201 (10)	0.2282 (2)	0.42108 (16)	0.0223 (5)	
H11A	0.9658	0.1603	0.4269	0.027*	
C12A	0.96711 (10)	0.3422 (2)	0.42520 (16)	0.0234 (5)	
H12A	1.0074	0.3508	0.4332	0.028*	
C13A	0.93159 (10)	0.4429 (2)	0.41722 (17)	0.0244 (5)	
H13A	0.9481	0.5195	0.4202	0.029*	
C14A	0.87147 (10)	0.4300 (2)	0.40483 (16)	0.0222 (5)	
H14A	0.8479	0.4982	0.4001	0.027*	
C15A	0.63902 (10)	−0.3212 (2)	0.38016 (16)	0.0217 (5)	
H15A	0.6036	−0.3371	0.3424	0.026*	
H15B	0.6402	−0.3775	0.4351	0.026*	
C16A	0.69204 (10)	−0.3417 (2)	0.31475 (17)	0.0250 (5)	
H16A	0.6865	−0.4152	0.2769	0.030*	
H16B	0.6956	−0.2753	0.2684	0.030*	
C17A	0.61045 (10)	−0.1931 (2)	0.51788 (16)	0.0222 (5)	
H17A	0.6280	−0.2564	0.5578	0.027*	
H17B	0.5689	−0.2110	0.5116	0.027*	
C18A	0.61714 (10)	−0.0742 (2)	0.57241 (17)	0.0225 (5)	
H18A	0.5957	−0.0124	0.5367	0.027*	
H18B	0.5995	−0.0819	0.6374	0.027*	
O1B	0.75199 (7)	0.42678 (15)	0.63678 (14)	0.0322 (4)	
O2B	1.00570 (7)	0.29674 (15)	0.68363 (12)	0.0273 (4)	
O3B	0.93973 (7)	0.43732 (15)	0.69510 (13)	0.0297 (4)	
O4B	0.75112 (7)	−0.22469 (16)	0.54787 (15)	0.0344 (4)	
HO4B	0.7437 (17)	−0.269 (4)	0.482 (3)	0.097 (14)*	
O5B	0.81384 (7)	−0.05141 (15)	0.39894 (13)	0.0241 (4)	
HO5B	0.7984 (15)	−0.080 (3)	0.455 (3)	0.066 (11)*	
N1B	0.74752 (7)	0.22275 (16)	0.62409 (13)	0.0172 (4)	
H1B	0.7240	0.1631	0.6150	0.021*	
N2B	0.95413 (8)	0.33162 (17)	0.68277 (13)	0.0207 (4)	
N3B	0.87113 (8)	−0.15414 (16)	0.55811 (13)	0.0189 (4)	
C1B	0.88035 (9)	0.0342 (2)	0.65113 (15)	0.0171 (5)	
C2B	0.82249 (9)	0.0722 (2)	0.63819 (15)	0.0175 (4)	
H2B	0.7933	0.0147	0.6286	0.021*	
C3B	0.80713 (9)	0.1941 (2)	0.63923 (14)	0.0164 (4)	
C4B	0.85090 (9)	0.2812 (2)	0.65427 (15)	0.0182 (5)	
H4B	0.8421	0.3631	0.6555	0.022*	
C5B	0.90790 (9)	0.2400 (2)	0.66727 (15)	0.0181 (5)	
C6B	0.92402 (9)	0.1200 (2)	0.66600 (15)	0.0182 (5)	
H6B	0.9630	0.0972	0.6748	0.022*	
C7B	0.89400 (10)	−0.0993 (2)	0.64869 (16)	0.0201 (5)	
H7B1	0.9361	−0.1111	0.6520	0.024*	
H7B2	0.8765	−0.1383	0.7058	0.024*	
C8B	0.72308 (9)	0.3357 (2)	0.62235 (16)	0.0183 (5)	
C9B	0.65804 (9)	0.3421 (2)	0.60460 (15)	0.0174 (5)	
C10B	0.62171 (9)	0.2426 (2)	0.59274 (16)	0.0211 (5)	
H10B	0.6376	0.1653	0.5935	0.025*	
C11B	0.56166 (10)	0.2582 (2)	0.57970 (17)	0.0230 (5)	
H11B	0.5375	0.1912	0.5718	0.028*	
C12B	0.53755 (10)	0.3727 (2)	0.57847 (16)	0.0230 (5)	
H12B	0.4973	0.3826	0.5699	0.028*	
C13B	0.57351 (10)	0.4725 (2)	0.59003 (17)	0.0244 (5)	
H13B	0.5574	0.5496	0.5894	0.029*	
C14B	0.63358 (10)	0.4578 (2)	0.60249 (16)	0.0217 (5)	
H14B	0.6576	0.5251	0.6095	0.026*	
C15B	0.85390 (10)	−0.2800 (2)	0.56934 (18)	0.0241 (5)	
H15C	0.8810	−0.3205	0.6138	0.029*	
H15D	0.8556	−0.3202	0.5056	0.029*	
C16B	0.79190 (10)	−0.2881 (2)	0.6105 (2)	0.0295 (6)	
H16C	0.7803	−0.3721	0.6152	0.035*	
H16D	0.7910	−0.2537	0.6765	0.035*	
C17B	0.90493 (9)	−0.1255 (2)	0.46962 (16)	0.0219 (5)	
H17C	0.9354	−0.1856	0.4603	0.026*	
H17D	0.9237	−0.0476	0.4777	0.026*	
C18B	0.86478 (10)	−0.1229 (2)	0.37859 (17)	0.0256 (5)	
H18C	0.8858	−0.0891	0.3227	0.031*	
H18D	0.8530	−0.2044	0.3616	0.031*	

### Atomic displacement parameters (Å^2^)

**Table:**

	*U*^11^	*U*^22^	*U*^33^	*U*^12^	*U*^13^	*U*^23^
O1A	0.0197 (8)	0.0177 (9)	0.0446 (10)	0.0000 (7)	−0.0021 (7)	−0.0027 (7)
O2A	0.0161 (8)	0.0301 (10)	0.0381 (10)	0.0037 (7)	−0.0001 (7)	0.0003 (8)
O3A	0.0264 (9)	0.0175 (9)	0.0500 (11)	0.0047 (7)	−0.0008 (8)	0.0038 (8)
O4A	0.0260 (9)	0.0202 (10)	0.0433 (10)	0.0004 (7)	−0.0023 (8)	−0.0025 (8)
O5A	0.0189 (8)	0.0200 (9)	0.0269 (9)	−0.0015 (7)	−0.0029 (6)	−0.0022 (7)
N1A	0.0124 (9)	0.0179 (10)	0.0203 (9)	0.0004 (7)	0.0003 (7)	0.0007 (7)
N2A	0.0177 (10)	0.0245 (11)	0.0231 (10)	0.0042 (8)	0.0009 (7)	−0.0007 (8)
N3A	0.0184 (9)	0.0159 (10)	0.0216 (9)	−0.0025 (7)	−0.0008 (7)	0.0015 (7)
C1A	0.0160 (11)	0.0223 (12)	0.0172 (11)	−0.0010 (9)	−0.0012 (8)	0.0007 (9)
C2A	0.0165 (11)	0.0197 (12)	0.0166 (10)	0.0022 (9)	−0.0004 (8)	0.0013 (8)
C3A	0.0140 (10)	0.0212 (12)	0.0148 (10)	−0.0024 (9)	0.0008 (8)	0.0000 (8)
C4A	0.0172 (11)	0.0184 (12)	0.0180 (11)	−0.0006 (9)	0.0019 (8)	0.0009 (8)
C5A	0.0162 (11)	0.0225 (12)	0.0171 (10)	0.0037 (9)	0.0016 (8)	0.0010 (9)
C6A	0.0134 (10)	0.0231 (12)	0.0174 (10)	−0.0027 (9)	0.0019 (8)	0.0011 (9)
C7A	0.0151 (10)	0.0193 (12)	0.0252 (12)	−0.0027 (9)	−0.0041 (9)	0.0002 (9)
C8A	0.0195 (11)	0.0184 (12)	0.0156 (10)	0.0003 (9)	0.0014 (8)	0.0006 (8)
C9A	0.0145 (10)	0.0248 (12)	0.0116 (10)	−0.0020 (9)	0.0011 (8)	−0.0003 (8)
C10A	0.0176 (11)	0.0198 (12)	0.0188 (11)	−0.0031 (9)	0.0007 (8)	0.0002 (9)
C11A	0.0191 (11)	0.0266 (13)	0.0213 (11)	0.0031 (10)	0.0001 (9)	0.0023 (9)
C12A	0.0152 (11)	0.0326 (14)	0.0223 (12)	−0.0056 (10)	−0.0007 (9)	0.0000 (10)
C13A	0.0216 (12)	0.0227 (13)	0.0290 (12)	−0.0079 (10)	0.0007 (10)	−0.0021 (10)
C14A	0.0205 (12)	0.0219 (12)	0.0242 (12)	−0.0008 (9)	−0.0004 (9)	−0.0022 (9)
C15A	0.0234 (12)	0.0193 (12)	0.0226 (11)	−0.0049 (9)	−0.0001 (9)	−0.0001 (9)
C16A	0.0262 (12)	0.0216 (13)	0.0271 (12)	0.0016 (10)	0.0019 (10)	−0.0004 (9)
C17A	0.0201 (11)	0.0217 (12)	0.0250 (12)	−0.0027 (9)	0.0020 (9)	−0.0009 (9)
C18A	0.0195 (11)	0.0245 (13)	0.0233 (12)	−0.0001 (10)	0.0031 (9)	−0.0017 (9)
O1B	0.0197 (9)	0.0183 (9)	0.0585 (12)	−0.0010 (7)	−0.0027 (8)	−0.0001 (8)
O2B	0.0166 (8)	0.0315 (10)	0.0340 (9)	−0.0032 (7)	0.0004 (7)	−0.0044 (8)
O3B	0.0248 (9)	0.0210 (10)	0.0432 (10)	−0.0052 (7)	0.0037 (8)	−0.0076 (7)
O4B	0.0218 (9)	0.0294 (10)	0.0519 (12)	0.0049 (8)	−0.0131 (8)	−0.0103 (9)
O5B	0.0194 (8)	0.0239 (9)	0.0289 (9)	0.0039 (7)	−0.0015 (7)	0.0039 (7)
N1B	0.0120 (9)	0.0178 (10)	0.0217 (9)	−0.0008 (7)	−0.0002 (7)	−0.0017 (7)
N2B	0.0178 (10)	0.0248 (11)	0.0195 (9)	−0.0052 (8)	0.0029 (7)	−0.0023 (8)
N3B	0.0186 (9)	0.0159 (10)	0.0223 (10)	0.0001 (7)	0.0000 (7)	−0.0012 (7)
C1B	0.0164 (11)	0.0211 (12)	0.0136 (10)	0.0001 (9)	−0.0007 (8)	0.0007 (8)
C2B	0.0171 (11)	0.0183 (11)	0.0170 (10)	−0.0029 (9)	−0.0002 (8)	−0.0019 (8)
C3B	0.0154 (10)	0.0214 (12)	0.0124 (10)	−0.0005 (9)	0.0017 (8)	−0.0020 (8)
C4B	0.0188 (11)	0.0191 (12)	0.0168 (10)	0.0013 (9)	0.0031 (8)	−0.0018 (8)
C5B	0.0174 (11)	0.0234 (12)	0.0134 (10)	−0.0053 (9)	0.0032 (8)	−0.0020 (8)
C6B	0.0142 (10)	0.0251 (12)	0.0154 (10)	0.0009 (9)	0.0005 (8)	−0.0023 (9)
C7B	0.0170 (11)	0.0194 (12)	0.0238 (11)	0.0026 (9)	−0.0029 (9)	0.0016 (9)
C8B	0.0190 (11)	0.0170 (12)	0.0189 (11)	0.0001 (9)	0.0012 (8)	0.0012 (8)
C9B	0.0168 (11)	0.0209 (12)	0.0145 (10)	0.0019 (9)	0.0018 (8)	0.0011 (8)
C10B	0.0187 (11)	0.0185 (12)	0.0260 (12)	0.0020 (9)	0.0003 (9)	−0.0020 (9)
C11B	0.0184 (11)	0.0246 (13)	0.0262 (12)	−0.0016 (10)	0.0007 (9)	−0.0012 (9)
C12B	0.0169 (11)	0.0306 (14)	0.0216 (11)	0.0041 (10)	−0.0006 (9)	0.0006 (10)
C13B	0.0255 (12)	0.0209 (13)	0.0267 (12)	0.0086 (10)	−0.0011 (10)	0.0016 (9)
C14B	0.0228 (12)	0.0191 (12)	0.0232 (11)	−0.0002 (9)	0.0003 (9)	0.0022 (9)
C15B	0.0229 (12)	0.0172 (12)	0.0322 (13)	0.0012 (10)	−0.0023 (10)	−0.0003 (9)
C16B	0.0217 (12)	0.0218 (13)	0.0449 (15)	0.0001 (10)	−0.0013 (11)	0.0010 (11)
C17B	0.0169 (11)	0.0228 (12)	0.0259 (12)	0.0029 (9)	0.0018 (9)	−0.0008 (9)
C18B	0.0261 (12)	0.0266 (13)	0.0243 (12)	0.0027 (10)	−0.0003 (10)	−0.0021 (10)

### Geometric parameters (Å, °)

**Table:**

O1A—C8A	1.232 (3)	O1B—C8B	1.221 (3)
O2A—N2A	1.238 (2)	O2B—N2B	1.237 (2)
O3A—N2A	1.225 (3)	O3B—N2B	1.229 (2)
O4A—C16A	1.440 (3)	O4B—C16B	1.439 (3)
O4A—HO4A	0.93 (4)	O4B—HO4B	1.03 (4)
O5A—C18A	1.433 (3)	O5B—C18B	1.433 (3)
O5A—HO5A	0.89 (3)	O5B—HO5B	0.90 (4)
N1A—C8A	1.357 (3)	N1B—C8B	1.371 (3)
N1A—C3A	1.419 (3)	N1B—C3B	1.410 (3)
N1A—H1A	0.8600	N1B—H1B	0.8600
N2A—C5A	1.471 (3)	N2B—C5B	1.478 (3)
N3A—C17A	1.465 (3)	N3B—C15B	1.458 (3)
N3A—C7A	1.472 (3)	N3B—C17B	1.458 (3)
N3A—C15A	1.481 (3)	N3B—C7B	1.462 (3)
C1A—C2A	1.394 (3)	C1B—C6B	1.391 (3)
C1A—C6A	1.396 (3)	C1B—C2B	1.394 (3)
C1A—C7A	1.506 (3)	C1B—C7B	1.514 (3)
C2A—C3A	1.396 (3)	C2B—C3B	1.396 (3)
C2A—H2A	0.9300	C2B—H2B	0.9300
C3A—C4A	1.401 (3)	C3B—C4B	1.403 (3)
C4A—C5A	1.387 (3)	C4B—C5B	1.388 (3)
C4A—H4A	0.9300	C4B—H4B	0.9300
C5A—C6A	1.381 (3)	C5B—C6B	1.380 (3)
C6A—H6A	0.9300	C6B—H6B	0.9300
C7A—H7A1	0.9700	C7B—H7B1	0.9700
C7A—H7A2	0.9700	C7B—H7B2	0.9700
C8A—C9A	1.503 (3)	C8B—C9B	1.503 (3)
C9A—C10A	1.396 (3)	C9B—C10B	1.389 (3)
C9A—C14A	1.398 (3)	C9B—C14B	1.399 (3)
C10A—C11A	1.383 (3)	C10B—C11B	1.390 (3)
C10A—H10A	0.9300	C10B—H10B	0.9300
C11A—C12A	1.389 (3)	C11B—C12B	1.384 (3)
C11A—H11A	0.9300	C11B—H11B	0.9300
C12A—C13A	1.383 (3)	C12B—C13B	1.386 (3)
C12A—H12A	0.9300	C12B—H12B	0.9300
C13A—C14A	1.387 (3)	C13B—C14B	1.388 (3)
C13A—H13A	0.9300	C13B—H13B	0.9300
C14A—H14A	0.9300	C14B—H14B	0.9300
C15A—C16A	1.515 (3)	C15B—C16B	1.522 (3)
C15A—H15A	0.9700	C15B—H15C	0.9700
C15A—H15B	0.9700	C15B—H15D	0.9700
C16A—H16A	0.9700	C16B—H16C	0.9700
C16A—H16B	0.9700	C16B—H16D	0.9700
C17A—C18A	1.517 (3)	C17B—C18B	1.532 (3)
C17A—H17A	0.9700	C17B—H17C	0.9700
C17A—H17B	0.9700	C17B—H17D	0.9700
C18A—H18A	0.9700	C18B—H18C	0.9700
C18A—H18B	0.9700	C18B—H18D	0.9700
			
C16A—O4A—HO4A	104 (2)	C16B—O4B—HO4B	112 (2)
C18A—O5A—HO5A	110 (2)	C18B—O5B—HO5B	107 (2)
C8A—N1A—C3A	127.10 (19)	C8B—N1B—C3B	126.87 (19)
C8A—N1A—H1A	116.5	C8B—N1B—H1B	116.6
C3A—N1A—H1A	116.5	C3B—N1B—H1B	116.6
O3A—N2A—O2A	123.19 (19)	O3B—N2B—O2B	123.43 (19)
O3A—N2A—C5A	119.14 (18)	O3B—N2B—C5B	118.97 (18)
O2A—N2A—C5A	117.67 (19)	O2B—N2B—C5B	117.59 (19)
C17A—N3A—C7A	112.73 (17)	C15B—N3B—C17B	115.88 (18)
C17A—N3A—C15A	111.18 (17)	C15B—N3B—C7B	113.99 (18)
C7A—N3A—C15A	109.31 (17)	C17B—N3B—C7B	114.08 (17)
C2A—C1A—C6A	119.1 (2)	C6B—C1B—C2B	119.2 (2)
C2A—C1A—C7A	120.9 (2)	C6B—C1B—C7B	121.68 (19)
C6A—C1A—C7A	119.81 (19)	C2B—C1B—C7B	119.12 (19)
C1A—C2A—C3A	121.4 (2)	C1B—C2B—C3B	121.9 (2)
C1A—C2A—H2A	119.3	C1B—C2B—H2B	119.1
C3A—C2A—H2A	119.3	C3B—C2B—H2B	119.1
C2A—C3A—C4A	119.95 (19)	C2B—C3B—C4B	119.32 (19)
C2A—C3A—N1A	116.78 (19)	C2B—C3B—N1B	117.30 (19)
C4A—C3A—N1A	123.26 (19)	C4B—C3B—N1B	123.4 (2)
C5A—C4A—C3A	117.0 (2)	C5B—C4B—C3B	117.2 (2)
C5A—C4A—H4A	121.5	C5B—C4B—H4B	121.4
C3A—C4A—H4A	121.5	C3B—C4B—H4B	121.4
C6A—C5A—C4A	124.3 (2)	C6B—C5B—C4B	124.4 (2)
C6A—C5A—N2A	118.23 (19)	C6B—C5B—N2B	118.33 (19)
C4A—C5A—N2A	117.5 (2)	C4B—C5B—N2B	117.3 (2)
C5A—C6A—C1A	118.21 (19)	C5B—C6B—C1B	118.1 (2)
C5A—C6A—H6A	120.9	C5B—C6B—H6B	121.0
C1A—C6A—H6A	120.9	C1B—C6B—H6B	121.0
N3A—C7A—C1A	115.71 (17)	N3B—C7B—C1B	110.62 (17)
N3A—C7A—H7A1	108.4	N3B—C7B—H7B1	109.5
C1A—C7A—H7A1	108.4	C1B—C7B—H7B1	109.5
N3A—C7A—H7A2	108.4	N3B—C7B—H7B2	109.5
C1A—C7A—H7A2	108.4	C1B—C7B—H7B2	109.5
H7A1—C7A—H7A2	107.4	H7B1—C7B—H7B2	108.1
O1A—C8A—N1A	122.1 (2)	O1B—C8B—N1B	122.3 (2)
O1A—C8A—C9A	120.6 (2)	O1B—C8B—C9B	121.2 (2)
N1A—C8A—C9A	117.29 (19)	N1B—C8B—C9B	116.54 (19)
C10A—C9A—C14A	118.73 (19)	C10B—C9B—C14B	119.3 (2)
C10A—C9A—C8A	124.1 (2)	C10B—C9B—C8B	124.6 (2)
C14A—C9A—C8A	117.1 (2)	C14B—C9B—C8B	116.1 (2)
C11A—C10A—C9A	120.1 (2)	C11B—C10B—C9B	120.1 (2)
C11A—C10A—H10A	119.9	C11B—C10B—H10B	119.9
C9A—C10A—H10A	119.9	C9B—C10B—H10B	119.9
C10A—C11A—C12A	120.8 (2)	C12B—C11B—C10B	120.4 (2)
C10A—C11A—H11A	119.6	C12B—C11B—H11B	119.8
C12A—C11A—H11A	119.6	C10B—C11B—H11B	119.8
C13A—C12A—C11A	119.4 (2)	C11B—C12B—C13B	119.8 (2)
C13A—C12A—H12A	120.3	C11B—C12B—H12B	120.1
C11A—C12A—H12A	120.3	C13B—C12B—H12B	120.1
C12A—C13A—C14A	120.3 (2)	C12B—C13B—C14B	120.1 (2)
C12A—C13A—H13A	119.9	C12B—C13B—H13B	119.9
C14A—C13A—H13A	119.9	C14B—C13B—H13B	119.9
C13A—C14A—C9A	120.6 (2)	C13B—C14B—C9B	120.2 (2)
C13A—C14A—H14A	119.7	C13B—C14B—H14B	119.9
C9A—C14A—H14A	119.7	C9B—C14B—H14B	119.9
N3A—C15A—C16A	111.96 (18)	N3B—C15B—C16B	110.18 (19)
N3A—C15A—H15A	109.2	N3B—C15B—H15C	109.6
C16A—C15A—H15A	109.2	C16B—C15B—H15C	109.6
N3A—C15A—H15B	109.2	N3B—C15B—H15D	109.6
C16A—C15A—H15B	109.2	C16B—C15B—H15D	109.6
H15A—C15A—H15B	107.9	H15C—C15B—H15D	108.1
O4A—C16A—C15A	111.69 (19)	O4B—C16B—C15B	110.8 (2)
O4A—C16A—H16A	109.3	O4B—C16B—H16C	109.5
C15A—C16A—H16A	109.3	C15B—C16B—H16C	109.5
O4A—C16A—H16B	109.3	O4B—C16B—H16D	109.5
C15A—C16A—H16B	109.3	C15B—C16B—H16D	109.5
H16A—C16A—H16B	107.9	H16C—C16B—H16D	108.1
N3A—C17A—C18A	115.01 (19)	N3B—C17B—C18B	110.30 (18)
N3A—C17A—H17A	108.5	N3B—C17B—H17C	109.6
C18A—C17A—H17A	108.5	C18B—C17B—H17C	109.6
N3A—C17A—H17B	108.5	N3B—C17B—H17D	109.6
C18A—C17A—H17B	108.5	C18B—C17B—H17D	109.6
H17A—C17A—H17B	107.5	H17C—C17B—H17D	108.1
O5A—C18A—C17A	113.93 (18)	O5B—C18B—C17B	109.84 (18)
O5A—C18A—H18A	108.8	O5B—C18B—H18C	109.7
C17A—C18A—H18A	108.8	C17B—C18B—H18C	109.7
O5A—C18A—H18B	108.8	O5B—C18B—H18D	109.7
C17A—C18A—H18B	108.8	C17B—C18B—H18D	109.7
H18A—C18A—H18B	107.7	H18C—C18B—H18D	108.2

### Hydrogen-bond geometry (Å, °)

**Table:**

*D*—H···*A*	*D*—H	H···*A*	*D*···*A*	*D*—H···*A*
O4A—HO4A···O1A^i^	0.93 (4)	1.82 (4)	2.736 (2)	168 (3)
O5A—HO5A···O4B	0.89 (3)	1.85 (3)	2.738 (2)	178 (3)
N1A—H1A···O5B	0.86	2.27	3.089 (2)	159
O5B—HO5B···O4B	0.90 (4)	2.30 (4)	3.130 (3)	153 (3)
O4B—HO4B···O4A	1.03 (4)	1.75 (4)	2.762 (3)	169 (3)
N1B—H1B···O5A	0.86	2.49	3.332 (2)	167

Symmetry codes: (i) *x*, *y*−1, *z*.
